# Bioactive Edible Films Based on Arrowroot Starch Incorporated with Cranberry Powder: Microstructure, Thermal Properties, Ascorbic Acid Content and Sensory Analysis

**DOI:** 10.3390/polym11101650

**Published:** 2019-10-11

**Authors:** Farayde Matta Fakhouri, Gislaine Ferreira Nogueira, Rafael Augustus de Oliveira, José Ignacio Velasco

**Affiliations:** 1 Centre Català del Plàstic, Dpt. of Materials Science and Metallurgy, Universitat Politècnica de Catalunya, Carrer Colom 114, E-08222 Terrassa, Spain; jose.ignacio.velasco@upc.edu; 2 Faculty of Engineering, Federal University of Grande Dourados, Dourados, MS 79804-970, Brazil; 3 School of Agricultural Engineering, University of Campinas, Campinas, SP 13083-875, Brazilaugustus@feagri.unicamp.br (R.A.d.O.)

**Keywords:** natural polymers, arrowroot starch, gelatin, cranberry, properties, microstructure, X-ray diffraction, differential scanning calorimetry, sensory analysis, food packaging

## Abstract

The growing global awareness about environmental preservation has stimulated the search for alternatives to replace conventional plastics made from fossil sources. One of the advantages is using polymers from renewable sources, such as starch and gelatin, which, in addition to being biodegradable, may also be edible. The incorporation of cranberry into a polymeric matrix can transfer bioactive composite films, colour and flavour to the film, which are characteristic of this fruit, expanding its application to fruit stripes or colourful coatings for specific foods. In this context, the aim of this work was to evaluate the influence of the incorporation of 0, 5, 15, 25, 35, 45 and 55% (solids mass/biopolymer mass) cranberry powder on the microstructure, thermal properties, ascorbic acid content and sensory analysis of gelatin and arrowroot starch films obtained by casting. Scanning electron microscopy (SEM) images showed that the incorporation of cranberry made the film surface rough and irregular. All films presented an X-ray diffraction pattern typical of a semicrystalline material. The glass transition temperature (T_g_) decreased when increasing the concentration of cranberry in films. All films with cranberry presented high ascorbic acid content and were well accepted by the tasters when sensory analysis was performed.

## 1. Introduction

The use of packaging is essential, as it plays a fundamental role in controlling the interactions between food and the environment, protecting and maintaining product quality, beyond its basic function of containing the food [1]. However, the polymers used in this industry is made from non-renewable synthetic materials, which, despite having excellent functional properties, are causing serious environmental problems due to the generation of high amounts of non-degradable solid waste in the environment [2].

One alternative to reducing the environmental impact is to use natural polymers for packaging rather than traditional petroleum-based polymers [1,2,3,4,5]. Biodegradable polymers are those which, when exposed to the bioactive environment, are degraded by the enzymatic action of living organisms (such as bacteria, yeasts, fungi) and converted at the end of the process to CO_2_, H_2_O and biomass under aerobic conditions and hydrocarbons, methane and biomass under anaerobic conditions [6].

In this regard, both starch and gelatin are natural polymers that have been widely used in the preparation of polymeric matrices for applications such as edible and biodegradable films and coatings in the food industry. In addition, these natural polymers are capable of forming odourless, tasteless, colourless and non-toxic matrices. Starch also has the advantage of being abundant, renewable and present in different forms depending on its origin [2,7].

Arrowroot (*Maranta arundinaceae* L.) starch presents good digestibility, gelling ability and high amylose content [8,9,10,11], desirable characteristics for the formulation of films with good technological properties [12].

Gelatin, a protein of animal origin, is obtained from collagen via acid or basic hydrolysis using a catalyst [13]; it has the ability to form thermo-reversible gels after heating, dissolution and cooling. The formation of gelatin gels involves ionic combinations between the amino and carboxyl groups of its amino acids, with the support of hydrogen bonds [14,15]. Blends of starch and gelatin have been studied for the development of edible films [15]. Composite films have the applicability of combining the benefits of each of the biopolymers used.

Studies on the production of edible films containing fruits (liquid or powder form) have showed that the addition of fruit into the film-forming solution results in a film with bioactive compounds, antimicrobial and antioxidant properties, colour and flavour characteristic of the fruit, expanding its application to fruit stripes as a source of nutritional compounds, or as colourful coatings for specific foods such as sushi; or yet, as active food packaging or partial substitutes for non-biodegradable plastic packaging [16,17,18,19].

Cranberry fruit (*Vaccinium macrocarpon* Aiton) is native to North America, and is abundant in bioactive compounds such as flavonoids, glycosides, anthocyanins, proanthocyanidins, organic acids, phenolic compounds [20] and ascorbic acid (high levels, that is, 200 mg/kg of berries) [21]. Anthocyanin pigments are mainly responsible for the pink and red colour of cranberries [22] and have been studied due to their potential to act as an antioxidant [23]. The sour taste of fresh cranberries is due to their content of organic acids such as hippuric acid and benzoic acid [24].

The antioxidant properties of cranberry are well documented in the literature, being in the row of common fruits, since it is the fruit with the highest antioxidant activity regarding total oxyradical elimination capacity, followed by apple, red grape, strawberry, peach, lemon, pear, banana, orange and pineapple [25]. Cranberries are also known for their proanthocyanidin compounds, in particular proanthocyanidin-A, which is associated with having antibacterial properties and potent antiadhesives [26]. The bioactive compounds of cranberry demonstrated the ability to inhibit *Escherichia coli*, *Helicobacter pylori*, *Listeria monocytogenes*, *Staphylococcus aureus* and *Salmonella typhimurium* [27,28,29].

The incorporation of cranberry into the arrowroot starch and gelatin polymeric matrix can confer the resulting film with modifications in the thermal, morphological, mechanical and sensorial properties, as well as in the bioactivity, conferring bioactive compounds with antioxidant and antimicrobial activity and expanding their application as intelligent packaging. In addition, films can function as a cranberry encapsulant as they can form a protective barrier to their bioactive compounds and further promote a controlled release of these compounds onto the food surface during storage, extending their shelf life [2,3,7,18]. The aim of this study was to develop edible films based on arrowroot starch and evaluate the influence of cranberry powder on the microstructure, thermal properties, ascorbic acid content and sensory analysis of these films for their application in food packaging.

## 2. Materials and Methods 

### 2.1. Materials

Gelatin type A (Leiner Davis Gelatin, Cotia, Brazil), cranberry (*Vaccinium macrocarpon*) powder (Herbarium, Colombo, Brazil) and glycerol (Synth, Diadema, Brazil). Arrowroot starch containing 15.24 ± 0.19% of water, 0.40 ± 0.03% of protein, 0.12 ± 0.01 % of fat, 0.33 ± 0.01% ash and 83.91 ± 0.76% of carbohydrates [30] and amylose content of 35.20 ± 1.63% [31,32]. The arrowroot was obtained in partnership with the Faculty of Agronomy, Federal University of Grande Dourados. All other reagents used for the analysis were presented at analytical grade.

### 2.2. Methods

#### 2.2.1. Film Preparation 

Films were obtained by the casting solvent technique. Gelatin and arrowroot starch solutions were prepared separately. For the production of the gelatin solution, 5 g of gelatin was hydrated in 100 mL of distilled water for 1 h. After that, this solution was heated at 80 °C for 10 min in a thermostatic bath (TECNAL, Piracicaba, Brazil), without agitation, to avoid the formation of bubbles. The starch solution was obtained by dispersing 3 g of arrowroot starch into 100 mL of distilled water and heating until reaching 80 °C in a thermostatic bath (TECNAL, Piracicaba, Brazil), with constant agitation for about 10 min. The solutions were mixed at volume ratios of 1:1 (gelatin type A solution/arrowroot starch solution) and plasticizer (glycerol) was incorporated in a concentration of 10% (0.5 g) in relation to the gelatin mass (w/w) and 20% (0.6 g) in relation to the starch mass (w/w) in the filmogenic solutions (w/w). This stirring was carried out gently in order to avoid the formation of bubbles in the sample, maintaining the natural pH of the solution [15]. 

The solutions were prepared by dispersing the cranberry powder into 100 mL of water in different concentrations: 0% (0 g), 5% (0.4 g), 15% (1.2 g), 25% (2.0 g), 35% (2.8 g), 45% (3.6 g) and 55% (4.4 g) weight of cranberry in relation to the weight of the macromolecules in the filmogenic solutions (8 g).

After, 20 mL of filmogenic solution was deposited into Plexiglas dishes (11.8 cm diameter) and dried at 25 °C for 24 h until they could be easily removed from the support. The films were conditioned at 25 °C and 52% of relative humidity for 48 h before their characterization.

#### 2.2.2. Visual Aspect and Microstructure

A visual test was performed to select films that were flexible and presented homogeneous colour. The morphological characteristics of the surface and cross section developed for films was observed in a scanning electron microscope (SEM) with X-ray energy dispersive (EDS) detector bench (model of SEM Leo 440i, model of EDS: 6070, Leo 440i-Leo Electron Microscopy/Oxford–Cambridge, England). The film sample was placed on a double-sided carbon-adhesive tape adhered to stub, submitted to the application of a gold layer (model K450, Sputter Coater EMITECH, Kent, UK) and observed in a scanning electron microscope operated at 10 kV.

#### 2.2.3. X-ray Diffractometry (XRD)

The diaphragms were obtained using an X-ray diffractometer, model X’Pert, Philips Analytical X Ray (Almelo, Netherlands), analysis conditions: Voltage and current: 40 kV and 40 mA, Scanning range: 2 theta from 5° to 40°, step: 0, 1° speed: 0.0166°/s.

#### 2.2.4. Differential Scanning Calorimetry (DSC)

For the analysis of the thermal properties of the films, a differential scanning calorimeter (DSC1, Mettler Toledo, Schwerzenbach, Switzerland) was used. 10 mg of film sample was weighed on a microanalytical scale (MX5-Mettler Toledo, Schwerzenbach, Switzerland) using an aluminium dish (40 μL). For reference, a standard aluminium was used. The sample was submitted to a heating program of 25 to 160 °C at the rate of 10 °C/min, in an inert atmosphere (50 mL/min of N_2_). When the temperature reached 160 °C, the sample was held for 5 min at this temperature. After this first scan, the measurement cells were cooled with liquid nitrogen to 25 °C, followed by a second heating sweep of 25 to 160 °C at a rate of 10 °C/min in an inert environment (50 mL/min of N_2_). The glass transition temperature (T_g_) was calculated as the baseline inflection point, caused by the discontinuity of specific heat of the sample.

#### 2.2.5. Determination of Ascorbic Acid Content

The determination of ascorbic acid content was performed by the method of Tillmans, with titration of the sample with standardized solution of 2,6-dichlorophenolindofenol [33]. Retention of ascorbic acid was calculated according to Equation (1):

(1) $$R=\frac{V\times F\times 100}{A}$$

where R is the amount of ascorbic acid in the film (mg/100 g of sample), V is the volume of Tillmans solution spent in the titration, F is the solution factor and A is the mL of the sample used. The analyses were performed in triplicate of the duplicate, and a total of 6 values were obtained.

#### 2.2.6. Sensory Analysis

The sensory evaluation of the edible films was performed by 56 untrained tasters. The number of tasters and the order of presentation of samples followed the design of [34], which considers the first-order, carry over effects. The acceptance tests were performed to assess the appearance, colour, flavour, taste and overall acceptance attributes. The film samples were cut into square format (2 cm × 2 cm) and served on a white plate on a white table, monadically and coded with three digits. In the sensory evaluations, the testers evaluated how much they liked or disliked the samples, through a hedonic scale of nine points with the corresponding extremes: “disliked extremely” (1) and “liked extremely” (9).

#### 2.2.7. Statistical Analysis

The results for the responses of the experimental design were evaluated using Statistica 9.0 software (StatSoft, South America). Significant differences were evaluated by analysis of variance (ANOVA) and the Tukey test at 5% level of significance, using SAS software (SAS 9.2, Cary, NC, USA).

## 3. Results and Discussion

### 3.1. Visual Aspects and Microstructure

**Figure 1:**
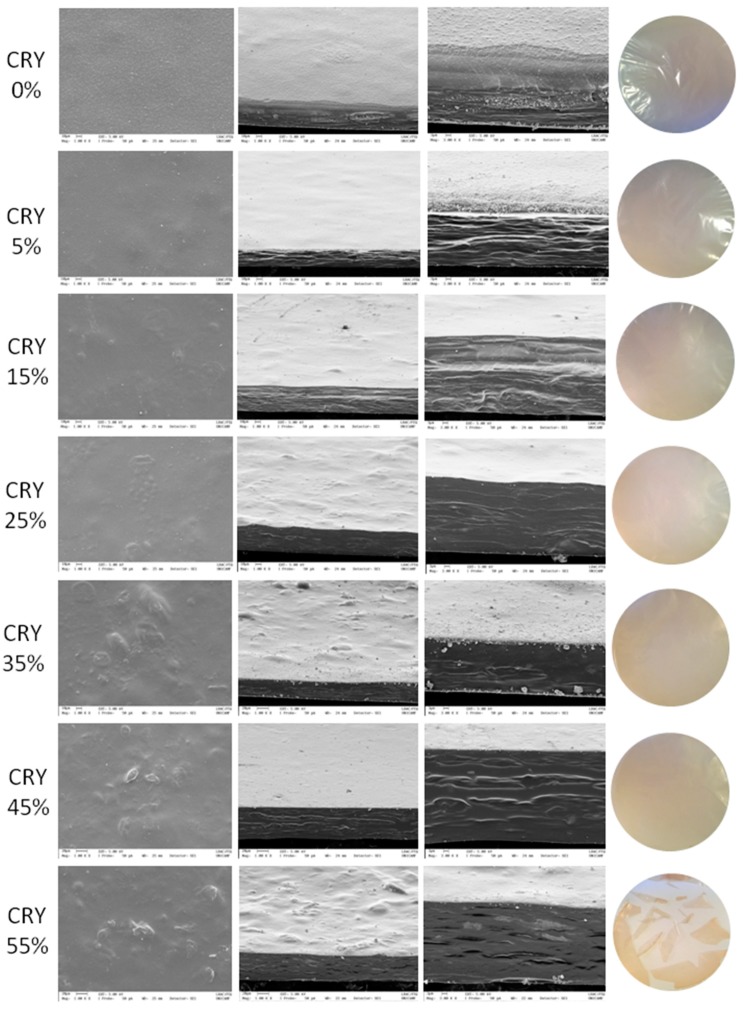
Scanning electron microscopy (SEM) images of surface (column 2, images with 1000× magnification), cross section (column 3, images with 1000× magnification, and column 4, images with 3000× magnification), and photographic images (column 5) of the edible films based on arrowroot starch and gelatine incorporated with 0, 5, 15, 25, 35, 45 and 55% cranberry.

Figure 1 shows the photography, SEM surface and cross section of the edible films based on arrowroot starch and gelatine incorporated with cranberry. Overall, the edible films made from arrowroot starch and gelatine incorporated with 0, 5, 15, 25, 35, 45 and 55% of cranberry were transparent.

Arrowroot starch and gelatine-based films were colourless and odourless, with a smooth surface and an organised polymer matrix. With the incorporation of cranberry powder, the film surface became rough, especially in films with 35, 45 and 55% of cranberry. This is due to the protuberances caused by suspended and agglomerated particles of cranberry powder in the polymer matrix. Similar characteristics were observed for films with blackberry [35,36], solid lipid microparticles containing ascorbic acid [7], lipid microparticles and starch nanoparticles [37]. The cross-section images of films showed multilayer structure, being more evident in films with 0, 5 and 15%, as well as it was for carrageenan-starch films with antioxidant extracts of Cuban red propolis and yerba mate prepared by casting [3].

The addition of cranberry at a higher concentration (55%) caused changes in the matrix of arrowroot starch and gelatine, generating cracks in the resulting film after the drying process. This confirms what was observed in the visual aspect, where difficulty in removing the films from the backing plates and difficulty in handling them, due to their more brittle appearance, had been reported. These characteristics suggest that this sample is more fragile than others in this study, and that the highest concentration of cranberry powder added to the films was 45%.

Films with cranberry showed a slightly pinkish coloration, especially at highest concentrations, evidencing the possible presence of anthocyanins. Anthocyanins are pigments responsible for the pink and red colour of cranberries [22]. Arrowroot starch films incorporated with blackberry also exhibited colour and flavour typical of blackberry powder, differing from films without blackberry, which were transparent and odourless [35]. This colour can be attractive for the development of food packages.

### 3.2. X-ray Diffractometry (XRD)

**Figure 2:**
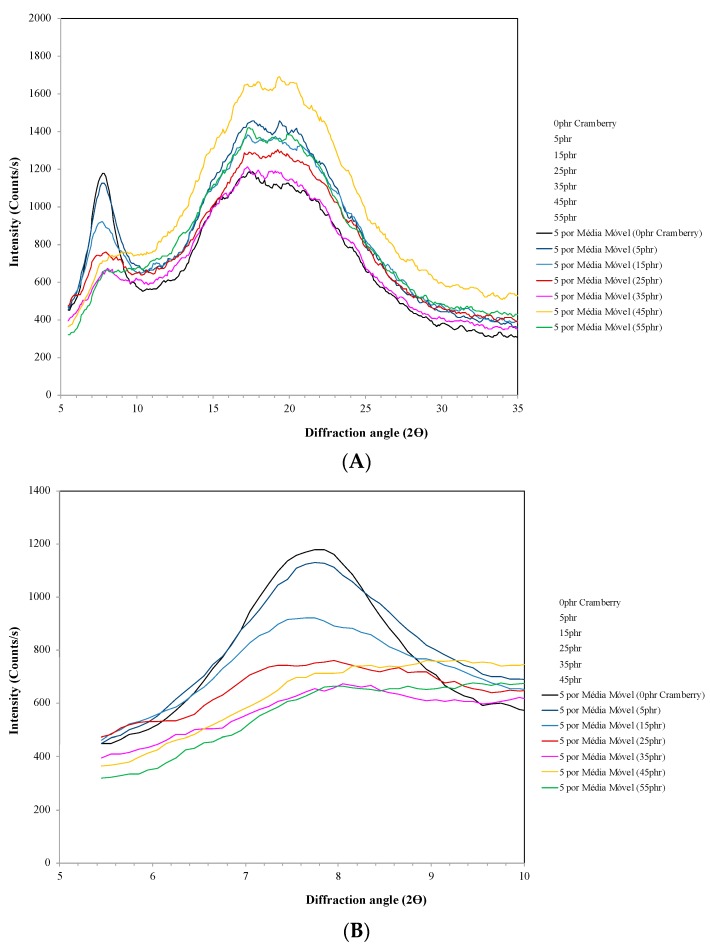
X-ray diffraction of arrowroot starch and gelatin films incorporated with cranberry, 0, 5, 15, 25, 35, 45 and 55%: (**A**) X-axis scale from 5 to 35; (**B**) X-axis scale from 5 to 10°.

The crystallinity of the arrowroot starch and gelatine films incorporated with 0, 5, 15, 25, 35, 45 and 55% of cranberry powder was evaluated by X-ray diffractometry; the diffractogram is shown in Figure 2A,B. It was possible to observe a pattern characteristic of a semi-crystalline material for the analysed samples. All films displayed an X-ray diffraction pattern typical of a partially crystalline material with two defined peaks: the first in the region of 2θ = 5° at 10°, with a maximum peak 2θ = 7.5°, corresponding to the crystalline triple helix structure of gelatine [38]; and the second in the region of 2θ = 12° at 30°, with maximum peak 2θ around 15 to 20°, being this behaviour typical of semi-crystalline polymers, such as starch and gelatine (Figure 2A). Arrowroot starch is a C-type crystallinity, characterized by main peaks at 2θ = 5.68, 15.42, 17.42°, being the most prominent the peak at 23.14° [8]. A-type gelatine has a crystalline XRD peak at 2θ = 20.9° [39]. In the diffractograms of films with cranberry (Figure 2B), it was also possible to verify that the intensity of the diffraction decreased at first peak and increased at second peak by increasing the concentration of cranberry incorporated into the arrowroot starch and gelatine film. This behaviour evidences a possible intermolecular interaction between the cranberry and the polymeric matrix of the film.

### 3.3. Differential Scanning Calorimetry (DSC)

**Figure 3:**
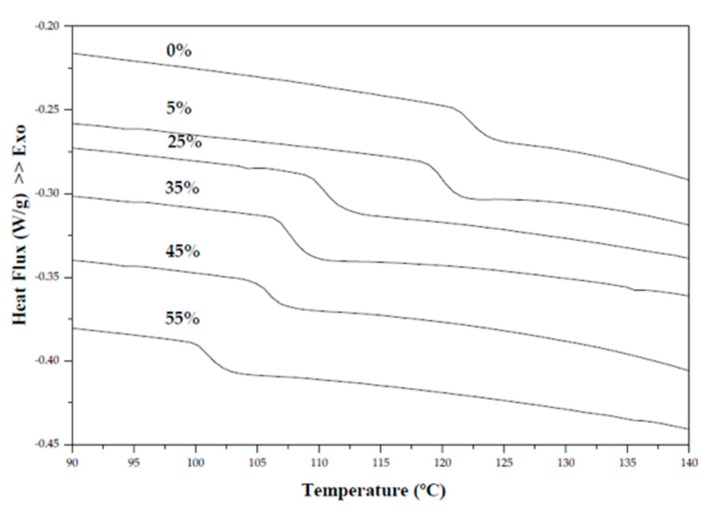
Differential scanning calorimetry (DSC) thermograms of edible films based on arrowroot starch and gelatin incorporated with cranberry.

DSC thermograms can be observed in Figure 3. The thermogram of films based on arrowroot starch and gelatin (0%) indicated the beginning of Tg around 121.2 °C; however, the glass transition temperature range decreased when increasing the concentration of cranberry by 5 to 55% in films, indicating Tg around 118.8 and 101.2 °C. Studies have reported that when fruit pulps are incorporated into the polymeric matrix of the film, the sugars found in the fruit will have a possible plasticizing effect [16,40]. In the film, the plasticizer acts by modifying the interaction between the polymers, leading to an increase in the free volume of the system, and consequently to an increase in chain motility and reduction of the glass transition temperature (T_g_) of the system [41,42].

### 3.4. Ascorbic Acid Content in Films

**Figure 4:**
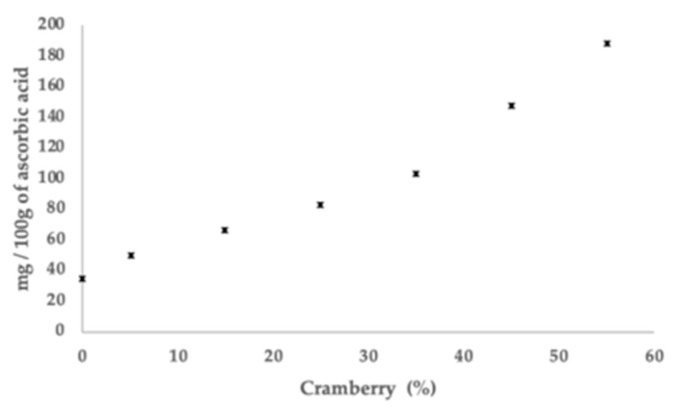
Ascorbic acid content of edible films from arrowroot starch and gelatin incorporated with cranberry.

Ascorbic acid is highly sensitive to heat and oxidation [43]. The incorporation of cranberry powder into the film can be a way to promote the protection of ascorbic acid. This is because the film can incorporate the active material into its protective matrix, essentially inert to the active material, thus acting as an encapsulant [44]. Consequently, the film matrix will act as a barrier for the protection against unfavourable environmental conditions (light, oxygen, pH, etc.), assisting in the stability of ascorbic acid. The values of 460 mg/100 g of ascorbic acid were found for cranberry powder and 33.88 ± 4.19 mg/100 g to 187.31 mg ± 7.91/100 g of ascorbic acid were found for films containing 0 and 55%, respectively (Figure 4). The results showed that increase the concentration of cranberry in the film-forming solution caused a significant increase in the ascorbic acid content embedded in the gelatin and arrowroot starch film. The ascorbic acid found in the film containing 0% may be derived from the ascorbic acid content present in the arrowroot starch (42.69 ± 5.75 mg/100 g) used as the basis for forming the films. It is possible that rhizomes, vitamins and minerals have been loaded together during the extraction of the starch from arrowroot.

### 3.5. Sensory Analysis

**Figure 5:**
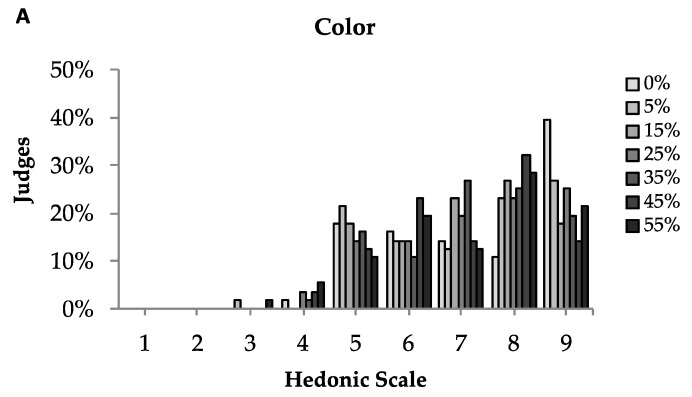

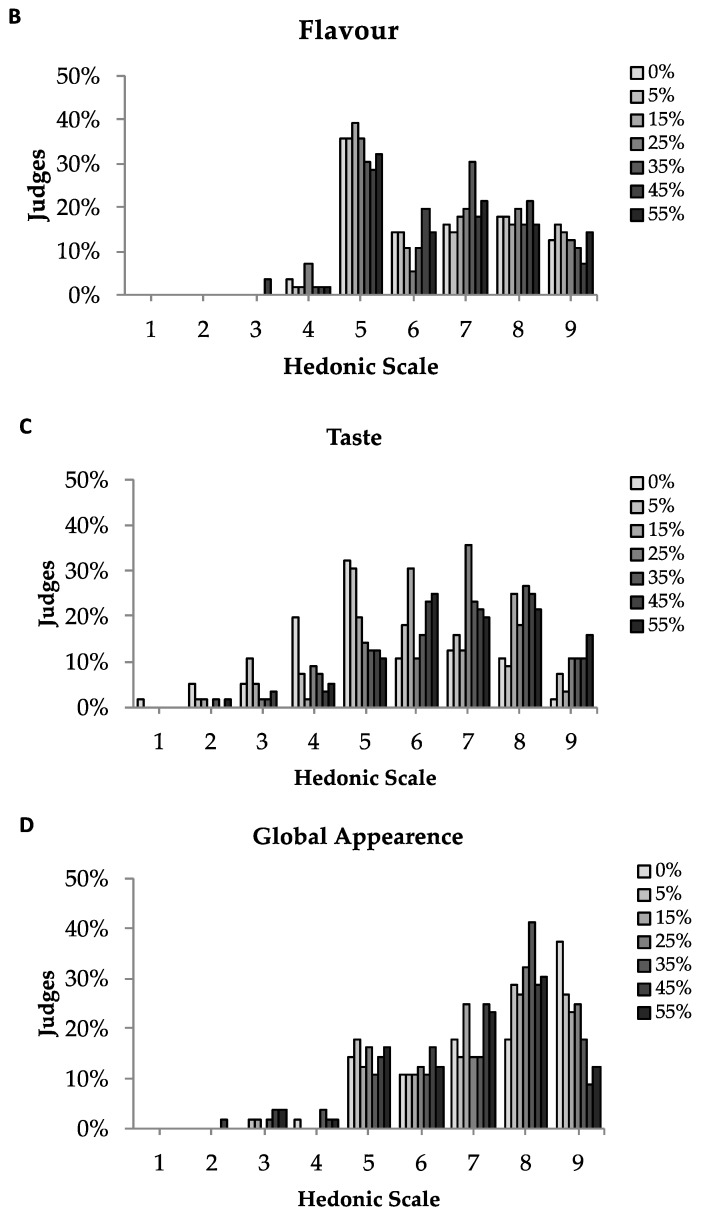
Sensory evaluation of films, using a hedonic scale (**A**) colour (**B**) flavour, (**C**) taste and (**D**) global acceptance. Mean of 56 consumers for each sample. Scores for global appearance: colour, flavour, taste and overall acceptance: 1 = disliked extremely; 2 = disliked very much; 3 = disliked moderately; 4 = disliked slightly; 5 = liked/disliked; 6 = liked slightly; 7 = liked moderately; 8 = liked very much; 9 = liked extremely.

The films were also very well accepted by the tasters when they were analysed, as shown in Table 1. The averages for all attributes evaluated were between 6 (slightly liked) and 7 (moderately liked) for most formulations of cranberry films, indicating high potential for their commercialization as edible packaging. For attributes such as appearance and colour, more than 77% of the testers rated very positive scores for the 7 film formulations between “liked slightly” to “liked extremely” (Figure 5). An important quality factor for dried foods is colour, since it reflects the sensory attractiveness [45], particularly for products like cranberry, which presents a striking reddish colour. This colour can be attractive for the development of certain food packages. With respect to the flavour, more than 57 and 61% of the tasters claimed to have enjoyed “slightly” and “extremely” the films with and without cranberry, respectively; only 7% remarked that they had slightly disliked the flavour of the film with 25% (cranberry powder). From the 56 tasters, only 36% rated grades ranging from “liked slightly” to “liked extremely” for the taste of the film with 0%, while 80% and 82% rated these grades for films with 45 and 55% cranberry, respectively. Cranberries are commonly consumed as fresh fruits, dried fruits or juices [46]. Cranberry fruit itself is very acidic and has a sour taste. This is due to their organic acids such as hippuric and benzoic acid [24]. The incorporation of cranberry into edible films can be a viable strategy for softening its sour taste and acidity and facilitate their commercialization. The results indicated that the incorporation of cranberry into the films improved its taste and, consequently, pleased the consumers; the same behaviour was observed for the global acceptance attribute.

## 4. Conclusions 

Films produced from arrowroot starch and gelatin incorporated with cranberry showed a decrease in T_g_ and were flexible for handling; no fractures or grooves were evident. The incorporation of cranberry into the film-forming solution in concentrations of 45 and 55% made it difficult to detach the films from the support plate. These high concentrations of cranberry may have restricted the molecular motility of the amylopectin chains, leading to an increase in T_g_, which made the films fragile and brittle for handling. SEM images showed that the incorporation of cranberry made the film surface rough and irregular. The X-ray diffraction of films revealed a partially crystalline structure. The incorporation of cranberry into the film-forming solution transferred ascorbic acid, colour and flavour to the resulting films, which may have led to a high acceptance of the films by the tasters when the sensory analysis was performed. The films proved to be nutritious because of the ascorbic acid content; they were well accepted by the tasters and could be a viable alternative to traditional packaging, being applied to fruit stripes as a source of nutritional compounds, or as colourful coatings for specific foods such as sushi; or as active food packaging or partial substitutes for non-biodegradable plastic packaging. However, new effects caused by this addition should be studied in other properties of the films.

## Figures and Tables

**Table 1:** Results of sensory evaluation averages.

Sample	Appearance	Colour	Flavour	Taste	Global Acceptation
0% CRY	7.48 ± 1.53	7.32 ± 1.63	6.46 ± 1.53	5.21 ± 1.74	6.09 ± 1.70
5% CRY	7.29 ± 1.56	7.13 ± 1.62	6.50 ± 1.54	5.71 ± 1.72	6.25 ± 1.49
15% CRY	7.30 ± 1.43	7.13 ± 1.36	6.58 ± 1.57	6.27 ± 1.57	6.61 ± 1.37
25% CRY	7.38 ± 1.41	7.20 ± 1.51	6.46 ± 1.61	6.66 ± 1.52	6.77 ± 1.57
35% CRY	7.27 ± 1.50	7.16 ± 1.40	6.60 ± 1.40	6.70 ± 1.64	6.71 ± 1.46
45% CRY	6.73 ± 1.59	7.02 ± 1.41	6.39 ± 1.50	6.73 ± 1.51	6.63 ± 1.52
55% CRY	6.91 ± 1.53	7.07 ± 1.61	6.60 ± 1.48	6.82 ± 1.57	6.89 ± 1.45

Sensory evaluation of films, using a hedonic scale Mean of 56 consumers for each sample. Scores for global appearance: colour, flavour, taste and overall acceptance: 1 = disliked extremely; 2 = disliked very much; 3 = disliked moderately; 4 = disliked slightly; 5 = liked/disliked; 6 = liked slightly; 7 = liked moderately; 8 = liked very much; 9 = liked extremely.
